# Humic Acid Derived from Vermicompost Inhibits Alveolar Bone Degradation and Protects Against Renal Injury in an Experimental Model of Periodontitis

**DOI:** 10.3390/biomedicines12122710

**Published:** 2024-11-27

**Authors:** Karen Rodrigues Lima, Hugo Giordano Tavares, Ramona Ramalho de Souza Pereira, Jaqueline do Carmo Lima Carvalho, Roberta de Oliveira Botelho, Aline Chaves Reis Spuri, Leonardo Barros Dobbss, Alan Rodrigues Teixeira Machado, Débora Ribeiro Orlando, Rafael Neodini Remédio, Saul Martins de Paiva, Rodrigo Ferreira de Moura, Marco Fabrício Dias-Peixoto, Luciano José Pereira, Eric Francelino Andrade

**Affiliations:** 1 Department of Health Sciences, Universidade Federal de Lavras (UFLA), Lavras 37200-000, MG, Brazil; karen.lima1@estudante.ufla.br (K.R.L.); roberta.botelho@estudante.ufla.br (R.d.O.B.); aline.reis@ufla.br (A.C.R.S.); debora.orlando@ufla.br (D.R.O.); rafael.remedio@ufla.br (R.N.R.); rodrigo.moura@ufla.br (R.F.d.M.); lucianojosepereira@ufla.br (L.J.P.); 2 Postgraduate Program in Health Sciences (PPGCS), Federal University of the Jequitinhonha and Mucuri Valleys (UFVJM), Diamantina 39803-371, MG, Brazil; hugo.giordano@ufvjm.edu.br (H.G.T.); ramona.souza@ufvjm.edu.br (R.R.d.S.P.); marcofabri@ufvjm.edu.br (M.F.D.-P.); 3 Department of Exact Sciences, Universidade do Estado de Minas Gerais, João Monlevade 35930-314, MG, Brazil; jaqueline.z.lima@gmail.com (J.d.C.L.C.); alan.machado@uemg.br (A.R.T.M.); 4 Institute of Agrarian Sciences, Universidade Federal dos Vales do Jequitinhonha e Mucuri (UFVJM), Unaí 38610-000, MG, Brazil; leonardo.dobbss@ufvjm.edu.br; 5 Department of Child and Adolescent Oral Health, Federal University of Minas Gerais, Belo Horizonte 31270-901, MG, Brazil; smpaiva@uol.com.br

**Keywords:** periodontitis, humic substances, bone, natural products

## Abstract

**Background:** Periodontal disease (PD) leads to the destruction of supportive tissues through an inflammatory response induced by biofilm accumulation. This low-grade systemic inflammation from PD increases the risk of comorbidities. Among potential therapeutic agents for PD, humic acids (HAs) are notable for their anti-inflammatory and immunomodulatory properties. This study aimed to evaluate the effects of varying HA doses on PD progression in an experimental model. **Methods:** Fifty-four Wistar rats were assigned to six groups (n = 8 each): control, PD, PD + 40 mg/kg HA, PD + 80 mg/kg HA, PD + 160 mg/kg HA, and PD + 320 mg/kg HA. HA from vermicompost was administered daily by gavage for 28 days, with PD induced by ligature on day 14. Post-euthanasia, mandibular samples were analyzed histomorphometrically for bone loss and osteocyte density. Alveolar bone topography and elemental composition were examined using Scanning Electron Microscopy (SEM) coupled with Energy Dispersive Spectroscopy (EDS). Renal and hepatic tissues were assessed histopathologically. Data were analyzed with Analysis of Variance (ANOVA) and Duncan’s test. **Results:** HA-treated animals showed reduced epithelial attachment loss and alveolar bone loss, with improved bone quality parameters, such as reduced pore number and diameter and increased osteocyte density compared to the PD group. Renal lesions observed in PD animals were mitigated at 40 and 80 mg/kg HA doses. **Conclusions:** HA treatment improves alveolar bone integrity and, at lower doses, reduces PD-induced renal lesions.

## 1. Introduction

Periodontal disease (PD) is characterized by the destruction of tooth-supporting tissues resulting from a negative interaction between oral bacteria and the host immune response, which leads to a self-perpetuating cycle of inflammation [1]. This oral dysbiosis initiates a chronic inflammatory response which, if untreated, can release inflammatory mediators into systemic circulation, affecting other tissues and compromising overall patient health [2]. Contributing factors such as obesity, excessive alcohol consumption, smoking, and aging exacerbate PD development and progression [3,4].

PD is a global public health concern, with severe cases increasing over recent decades [5]. In addition to its impact on oral health, PD is known to affect other systems, including hepatic and renal systems, underscoring the importance of its treatment and prevention. Periodontal therapy aims to control the inflammatory response, slow disease progression, and restore the structure and function of periodontal tissues [6,7]. The primary treatment for PD involves scaling and root planing. In severe cases, adjunctive therapies, such as laser treatments, antiseptics, and systemic antibiotics (e.g., amoxicillin and metronidazole), are also recommended [8,9]. In patients with advanced PD, surgical interventions are necessary to reduce or eliminate periodontal pockets [10]. Additionally, various strategies employing anti-inflammatory and/or immunomodulatory agents have been utilized to mitigate PD progression or prevent its onset, supporting both local inflammation control and host response to periodontal pathogens [1,11].

In this context, humic acids (HAs) hold considerable potential for PD treatment due to their demonstrated anti-inflammatory and antioxidant activities in other inflammatory diseases [12]. HAs are compounds found within humic substances, formed through the decomposition of organic matter from animal and plant organisms. Their chemical structure is rich in phenolic groups, which confer detoxifying, anti-inflammatory, and antioxidant properties [13]. The extraction of HA is typically carried out following the standard protocol established by the International Society of Humic Substances [12]. These compounds are a fraction of organic matter that is insoluble in acidic media. They are isolated through precipitation by lowering the pH of a solution containing humic substances (HA and fulvic acids) to a range of 1.5 to 2.0 during the fractionation process of organic matter. Studies evaluating HA in animal models have shown low toxicity, with no adverse effects observed even after 91 days of administration [14,15]. In the aforementioned study [14], no toxicity-related alterations were observed following treatment with an (HA)-containing compound. This included assessments of behavioral aspects, feed efficiency, clinical pathology parameters, and both absolute and relative organ weights. Furthermore, no histopathological changes were detected in the liver, kidneys, lungs, or stomach of male and female Wistar rats.

Moreover, HA has been reported to support bone regeneration by accelerating remineralization and enhancing osteoblast activity [16]. In a recent study by our group, PD-induced animals treated with 80 mg/kg of HA demonstrated reduced alveolar bone loss (ABL) compared to untreated controls [12]. However, despite existing evidence on HA’s effects in PD, further investigation is needed to understand the dose-dependent effects of this agent, both locally and on systemic functions such as hepatic and renal health. Therefore, this study aimed to evaluate the impact of various HA doses on alveolar bone and hepatic and renal integrity in rats with ligature-induced PD.

## 2. Materials and Methods

This study was approved by the Animal Use Ethics Committee of the Federal University of Jequitinhonha and Mucuri Valleys (CEUA/UFVJM—protocol 022/2021). All procedures were conducted following the Guide for the Care and Use of Laboratory Animals and ARRIVE guidelines. This preclinical study was conducted in the state of Minas Gerais, Brazil, with the in vivo study taking place between April and June of 2023.

### 2.1. Animals

Fifty-four healthy male Wistar rats (*Rattus norvegicus albinus*), eight weeks old and with an initial body weight of 360 ± 17 g, were used. The animals were sourced from the Central Animal Facility of the Federal University of Viçosa. Throughout the experiment, the rodents were housed in the experimental room under controlled temperature (22 ± 2 °C), humidity (45 ± 15%), and a 12 h light/dark cycle. Animals were kept in polyethylene cages lined with wood shavings and provided with rodent chow (Nuvilab^®^, Seoul, Republic of Korea) and water ad libitum for the entire experimental period.

### 2.2. Experimental Design

After a seven-day acclimation period, animals were randomly assigned to six experimental groups based on treatment (n = 8/group): control group (C)—animals without PD treated with saline solution; PD group—animals with PD treated with saline; PD + HA-40 group—animals with PD treated with 40 mg/kg of HA; PD + HA-80 group—animals with PD treated with 80 mg/kg of HA; PD + HA-160 group—animals with PD treated with 160 mg/kg of HA; and PD + HA-320 group—animals with PD treated with 320 mg/kg of HA.

HA was administered daily by gavage for 28 days, and on the 14th day of treatment, animals in the PD groups underwent ligature-induced PD. In the C and PD groups, 0.3 mL of saline solution was administered daily. At the end of the experimental period, animals were anesthetized with intraperitoneal injections of xylazine hydrochloride (10 mg/kg) and ketamine hydrochloride (80 mg/kg) and then euthanized via cardiac puncture.

### 2.3. Composting, Vermicomposting, Extraction, and Characterization of Humic Acids (HA)

Different agricultural biomasses were utilized for composting. Residues from soybean (*Glycine max* L.) and sorghum (*Sorghum bicolor* (L.) Moench), coarsely chopped, as well as corn grain flour (*Zea mays* L.) and crushed sugarcane bagasse (*Saccharum officinarum* L.) were composted for 30 days. The process included mechanical turning every 10 days. Subsequently, earthworms (*Eisenia foetida*) were added at a density of 50 per kilogram of organic material to initiate the composting process, which lasted approximately three months (95 days). Humidity, temperature, pH, and aeration parameters were monitored every three days. At the end of the process, the vermicompost was dried in an oven at 60 °C for 48 h, sieved through a 4 mm mesh, and used for the extraction of HA.

The extraction process began by treating the vermicompost with a 0.1 mol L⁻^1^ NaOH solution in a ratio of 1:10 (m/v) for four hours under agitation. After this period, the material was centrifuged (15 min at 5000 g) to separate the humic substances (humic acids—HAs + fulvic acids—FAs) from the humins (the insoluble fraction of organic matter). To separate the HAs from the FAs, the pH of the humic substances was reduced to between 1.0 and 1.5 by adding 6 mol L^−1^ HCl. At this pH, the HAs precipitated and were subsequently separated and washed with distilled water until a negative test for the presence of chloride was confirmed using silver nitrate (AgNO₃). After washing, the HAs were titrated to a pH of 7.0 using 0.01 mol L^−1^ KOH and then placed in membranes with a molecular weight cut-off of 1000 Da and dialyzed against distilled water until the electrical conductivity (EC) of the system reached equilibrium. Following dialysis, the HAs were frozen and lyophilized for later use. The extraction of HA was performed according to the standard protocol of the International Society of Humic Substances [12]. In all experimental stages conducted in the present study, approximately 4 g of humic acids was used, which was obtained from 800 g of vermicompost.

The morphology and chemical composition of the HAs were evaluated using a Scanning Electron Microscope (Vega 3 LMU, TESCAN, Brno-Kohoutovice, Czech Republic) equipped with an Energy Dispersive X-ray Spectroscopy (EDS) detector (X-MaxN, Oxford Instruments, Oxford, UK). The sample was deposited on carbon adhesive tape, fixed onto a stub, and coated with a thin layer of gold–palladium using a sputter coater (SC7620, Quorum Technologies, Ashford, UK).

### 2.4. Histomorphometric Analyses: Assessment of Attachment Loss and Alveolar Bone Loss

After euthanasia, the mandibles of Wistar rats, which had undergone PD induction for 14 days, were removed and bisected along the midline. The right hemimandible was used to assess alveolar bone loss (ABL) through histomorphometric analysis. A phosphate-buffered saline (PBS)–formaldehyde solution was employed to fix the tissues while preserving their integrity. The specimens were then subjected to routine histological processing [17]. For the preparation of histological slides, three serial sections with a thickness of 5 µm were obtained and stained with hematoxylin and eosin (H&E). The slides were photographed using a photomicroscope and analyzed with Leica Application Suite (LAS EZ) software (https://www.leica-microsystems.com/products/microscope-software/p/leica-las-ez/downloads/).

Alveolar bone loss (ABL) was evaluated beneath the first molar, as well as epithelial insertion loss between the first and second molars. To assess ABL, the distance from the cemento-enamel junction (CEJ) to the alveolar bone crest was measured, while the distance between the CEJ and the junctional epithelium was measured for epithelial insertion loss [18]. The average measurement of the three roots was utilized to quantify ABL. These parameters were evaluated using Image J software Version 1.54 (Bethesda, MD, USA). All measurements were conducted by a trained evaluator who was blinded to the experimental groups.

### 2.5. Assessment of Osteocyte Density

The density of osteocytes (number of osteocytes per area) in the region of the alveolar bone crest was evaluated in slides stained with hematoxylin and eosin. Three representative photographs were taken for each animal in each experimental group at a magnification of 40×, as previously described [19]. For each sample (three sections per animal), three random microscopic fields were analyzed to determine the number of osteocytes per area. The average osteocyte density was expressed as n/µm^2^.

### 2.6. Assessment of Alveolar Bone Loss and Morphological Analysis of Mandibular Bone Composition and Topography Using Scanning Electron Microscopy Coupled with Energy Dispersive X-Ray Spectroscopy (SEM/EDS)

The left hemimandibles were immersed in hydrogen peroxide for 12 h to facilitate the removal of remaining soft tissues and were subsequently dried at room temperature for two weeks. To evaluate the bone microstructure and identify any morphological changes in the mandibular bone, each sample was placed on the surface of an aluminum support using double-sided carbon tape and analyzed via Scanning Electron Microscopy (SEM) (Vega 3 LMU, TESCAN, Brno-Kohoutovice, Czech Republic). Assessments were conducted on the buccal surface beneath the first mandibular molar, with images obtained at magnifications of 27× and 500×.

For the images captured at 27× magnification, linear measurements were made of the distance between the cemento-enamel junction (CEJ) and the alveolar bone crest (ABC), as well as the area using the buccal surface [20]. The average of three linear measurements from each sample was used to express alveolar bone loss (ABL). The measurements of the area of resorption were evaluated blindly by a trained examiner (KRL).

Furthermore, the topography of the alveolar bone was qualitatively assessed in images at 500× magnification, considering aspects such as porosity, irregularities, and roughness beneath the first molar, as previously described [21]. A quantitative evaluation of the area and diameters of the pores was also conducted by selecting a standardized area of interest (60.59 × 10^3^ µm^2^) beneath the first mandibular molar in the upper-right quadrant. These analyses were performed by a trained, calibrated, and blinded examiner (ROB). Measurements were taken using Image J software Version 1.54 (Bethesda, MD, USA).

The elemental compositions (calcium, phosphorus, carbon, and oxygen) were determined using Energy Dispersive X-ray Spectroscopy (EDS) with an X-MaxN detector (Oxford Instruments, Abingdon, UK). Spectra were collected at an acceleration voltage of 20 kV with a working distance of 13 mm, and data analysis was performed using AZtec 3.1 software (Oxford Instruments) [20].

### 2.7. Histopathological Evaluation of Liver and Kidney Tissues via Optical Microscopy

Following euthanasia, fragments of renal and hepatic tissues were fixed in 10% buffered formalin for 48 h and subsequently underwent routine histological processing to prepare histological slides stained with hematoxylin and eosin. Three serial sections from each tissue of every animal were qualitatively analyzed by an experienced pathologist (DRO). Aspects such as tissue integrity and the presence of lesions were assessed using optical microscopy at a magnification of 40×. After the individual analysis of each sample, representative slides for each experimental group were photographed.

### 2.8. Statistical Analyses

The data were subjected to the Shapiro–Wilk test for normality and analyzed using One-Way Analysis of Variance (ANOVA) with a 95% confidence interval for multiple comparisons. In cases where significant differences were observed between treatments, Duncan’s Multiple Range Test was conducted. Data analysis was performed using GraphPad Prism 8 software.

## 3. Results

### 3.1. Scanning Electron Microscopy Analysis of Humic Acid (SEM/EDS)

**Figure 1:**
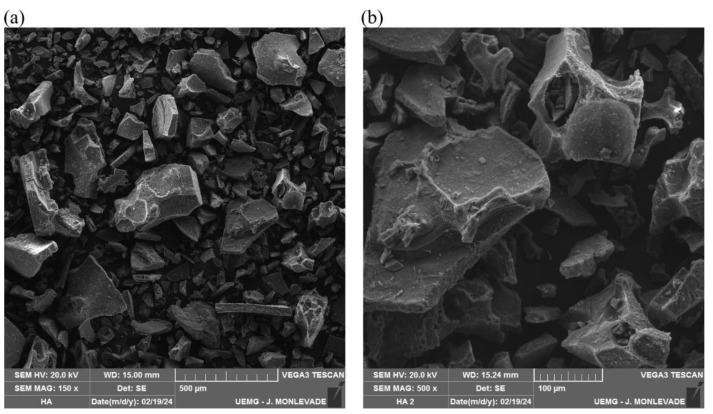
Electron scanning micrographs (SEM) of the humic acid isolated from vermicompost with magnification (**a**) of 150× and (**b**) of 500×.

SEM/EDS analysis of HA revealed a surface composed of compacted microaggregates (Figure 1). The primary elements in humic acid were identified using Energy Dispersive X-ray Spectroscopy (EDS), as shown in Table 1. As expected, the major elements detected were carbon (C) and oxygen (O). Additionally, smaller proportions of sodium (Na), bromine (Br), silicon (Si), potassium (K), calcium (Ca), iron (Fe), and titanium (Ti) were observed. Importantly, no heavy metals were detected in the sample, ensuring the evaluated HA’s safety and suitability for its intended applications.

### 3.2. Histomorphometric Analyses

**Figure 2:**
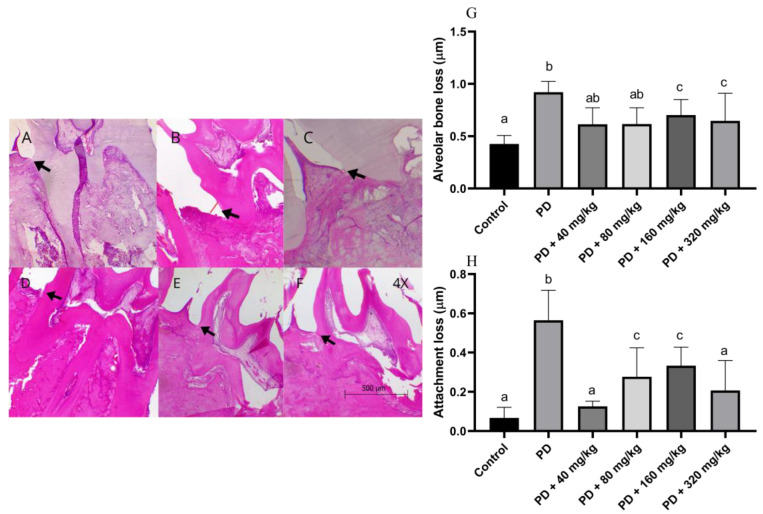
Assessment of alveolar bone loss and epithelial attachment loss in Wistar rats with ligature-induced periodontal disease treated with humic acid. (**A**–**F**). Arrows indicate the position of the cemento-enamel junction, either separated from or adjacent to the junctional epithelium, with the red line marking the space between the two structures. Magnification: 4×. (**A**): Control group. (**B**): Periodontal disease (PD) group. (**C**): PD + 40 mg/kg HA. (**D**): PD + 80 mg/kg HA. (**E**): PD + 160 mg/kg HA. (**F**): PD + 320 mg/kg HA. (**G**): Alveolar bone loss. (**H**): Attachment loss. Statistical analysis was conducted using ANOVA with Bartlett’s post hoc test (*p* < 0.05). Different letters (a–c) indicate statistically significant differences between experimental groups.

Histomorphometric analyses (Figure 2A–F) indicated that animals in the PD group not treated with HA exhibited greater epithelial attachment loss and ABL compared to the control group (Figure 2G,H). The mean ABL values were 0.41 ± 0.06 mm and 0.92 ± 0.08 mm for the control and PD groups, respectively. For epithelial attachment loss, the values were 0.05 ± 0.01 mm and 0.51 ± 0.11 mm, respectively. Additionally, animals treated with HA at all dosage levels showed significantly lower values for these parameters compared to the PD group (*p* < 0.05). We observed that the lowest ABL values among the treatments were found at the 40 mg/kg dose (0.61 ± 0.07 mm), while the highest values among the treated groups were observed at the 160 mg/kg dose (0.70 ± 0.10 mm). For epithelial attachment loss, the lowest values were also observed at the 40 mg/kg dose (0.13 ± 0.02 mm), whereas the highest values were recorded at the 160 mg/kg dose (0.33 ± 0.05 mm).

### 3.3. Osteocyte Density

**Figure 3:**
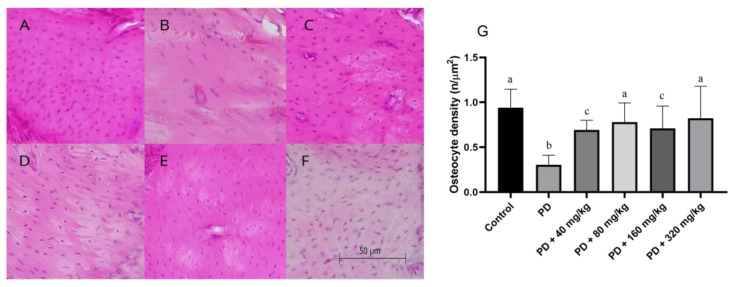
Osteocyte density per bone area beneath the first mandibular molar in Wistar rats with ligature-induced periodontal disease treated with humic acid. (**A**–**F**). Representation of osteocyte density according to experimental treatment. Magnification: 40×. (**A**): Control group. (**B**): Periodontal disease (PD) group. (**C**): PD + 40 mg/kg HA. (**D**): PD + 80 mg/kg HA. (**E**): PD + 160 mg/kg HA. (**F**): PD + 320 mg/kg HA. (**G**): Osteocyte density. Statistical analysis was conducted using ANOVA with Bartlett’s post hoc test (*p* < 0.05). Different letters (a–c) indicate statistically significant differences between experimental groups.

Figure 3A–F illustrate the osteocyte density observed across the experimental groups. Animals in the PD group showed significantly lower osteocyte density compared to the control group (*p* < 0.05; Figure 3). Animals treated with HA demonstrated higher osteocyte density compared to those in the PD group (*p* < 0.05; Figure 3G). Compared to the control group, osteocyte density did not differ at doses of 80 and 320 mg/kg (*p* > 0.05; Figure 3G). However, at doses of 40 and 160 mg/kg, the values for this parameter were significantly lower (*p* < 0.05; Figure 3G).

### 3.4. Alveolar Bone Loss (ABL) Assessment via SEM Images

**Figure 4:**
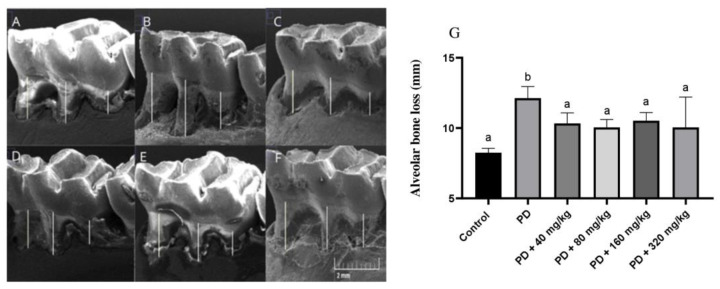
Alveolar bone loss (ABL) assessed in Scanning Electron Microscopy (SEM) images. ABL was evaluated in SEM images by measuring the distance between the cemento-enamel junction and the alveolar bone crest of the first mandibular molar roots in Wistar rats with ligature-induced periodontal disease treated with humic acid. Panels (**A**–**F**). Representation of the region of interest where measurements were taken. Magnification: 27×. (**A**): Control group. (**B**): Periodontal disease (PD) group. (**C**): PD + 40 mg/kg HA. (**D**): PD + 80 mg/kg HA. (**E**): PD + 160 mg/kg HA. (**F**): PD + 320 mg/kg HA. (**G**): Alveolar bone loss. Statistical analysis was conducted using ANOVA with Bartlett’s post hoc test (*p* < 0.05). Different letters (a,b) indicate statistically significant differences between experimental groups.

Figure 4A–F represent ABL assessed in SEM images. ABL was significantly greater in animals from the PD group compared to the control group (*p* < 0.05—Figure 4). Additionally, ABL was attenuated in all groups treated with HA (*p* < 0.05; Figure 4G). Compared to the control group, the ABL values were higher in all groups treated with HA (*p* < 0.05; Figure 4).

### 3.5. Elemental Composition of the Alveolar Bone Surface

**Figure 5:**
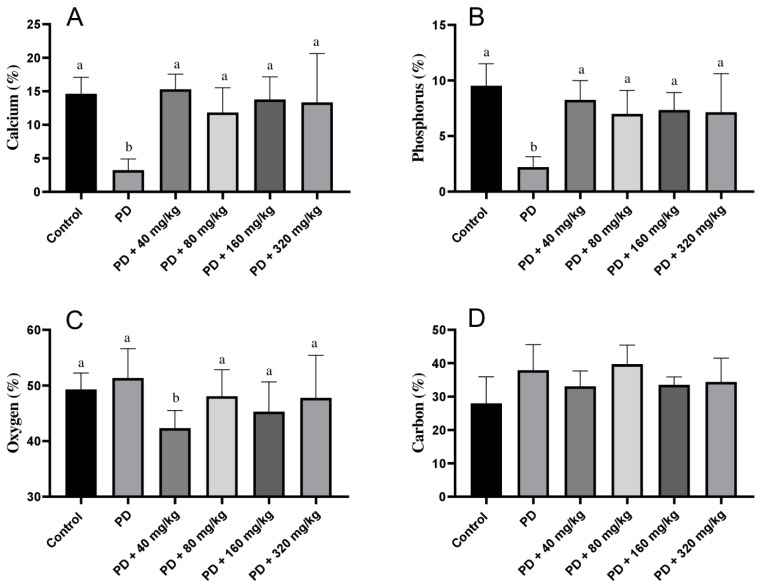
Elemental composition of alveolar bone beneath the first mandibular molar in Wistar rats with ligature-induced periodontal disease treated with humic acid. (**A**): Calcium percentage in bone. (**B**): Phosphorus percentage in bone. (**C**): Oxygen percentage in bone. (**D**): Carbon percentage in bone. Statistical analysis was conducted using ANOVA with Bartlett’s post hoc test (*p* < 0.05). Different letters (a,b) indicate statistically significant differences between experimental groups.

Regarding the elemental composition of the alveolar bone surface, we observed that calcium and phosphorus percentages were lower in animals with PD compared to the control group (*p* < 0.05). Animals treated with HA showed higher levels of these elements than the PD group (*p* < 0.05), with values comparable to those observed in the control group (*p* > 0.05; Figure 5).

### 3.6. Topography of Alveolar Bone

**Figure 6:**
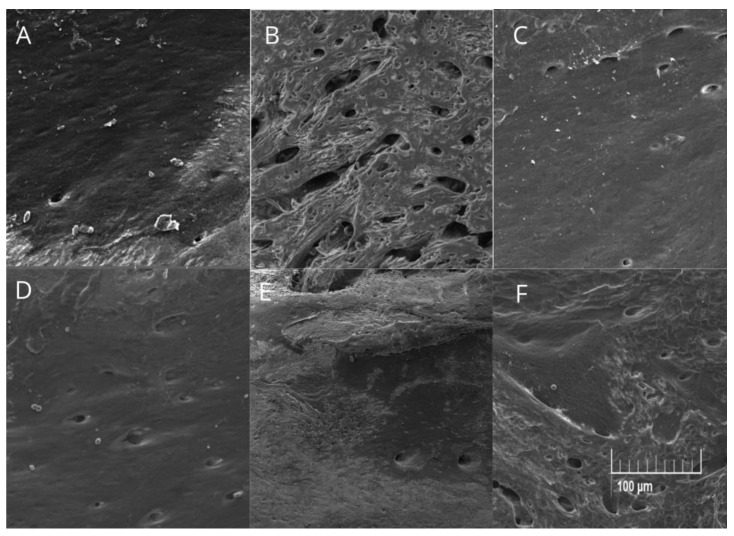
Representative images of the topography of the alveolar bone area beneath the first mandibular molar in Wistar rats with ligature-induced periodontal disease treated with humic acid (HA). Magnification: 500× in Scanning Electron Microscopy (SEM). (**A**): Control group. (**B**): Periodontal disease (PD) group. (**C**): PD + 40 mg/kg HA. (**D**): PD + 80 mg/kg HA. (**E**): PD + 160 mg/kg HA. (**F**): PD + 320 mg/kg HA.

In the qualitative assessment of the porosity and roughness of the alveolar bone beneath the first mandibular molar, it was observed that animals in the PD group exhibited greater irregularities, porosity, and roughness compared to the other experimental groups (Figure 6).

### 3.7. Quantitative Analysis of Alveolar Bone Porosity Beneath the First Mandibular Molar

**Figure 7:**
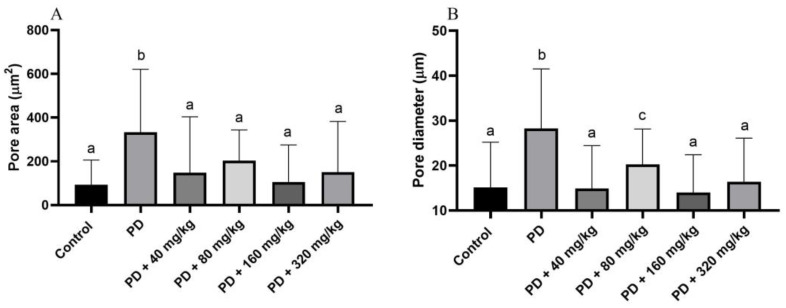
Area (**A**) and diameter (**B**) of pores on the surface of alveolar bone beneath the first mandibular molar in Wistar rats with ligature-induced periodontal disease treated with humic acid (HA). Statistical analysis was conducted using ANOVA with Bartlett’s post hoc test (*p* < 0.05). Different letters (a–c) indicate statistically significant differences between experimental groups.

In the quantitative analysis of the porosity of the alveolar bone beneath the first molar, assessed through SEM images, it was observed that all treated groups exhibited smaller pores, both in area (*p* < 0.05; Figure 7A) and in diameter (*p* < 0.05; Figure 7B), compared to the PD group. No differences were observed in pore area between the HA-treated groups and the control group (*p* > 0.05; Figure 7A). Similarly, no significant differences in pore diameter were found between the control group and the HA-treated groups at doses of 40, 160, and 320 mg/kg (*p* > 0.05; Figure 7A). However, animals treated with HA at a dose of 80 mg/kg exhibited significantly larger pore diameters compared to the control group (*p* < 0.05; Figure 7A).

### 3.8. Histopathological Evaluation of Liver and Kidney Findings

**Figure 8:**
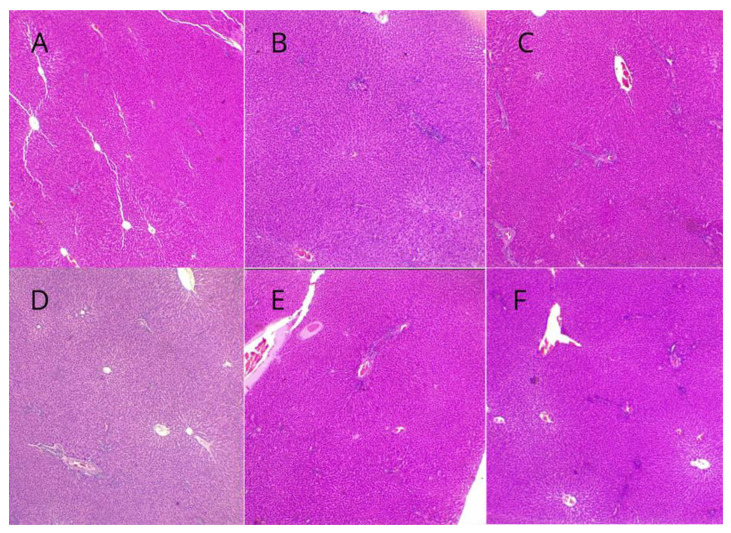
Representative images of hepatic tissue without alterations from the experimental groups. Magnification: 4×. (**A**): Control group. (**B**): Periodontal disease (PD) group. (**C**): PD + 40 mg/kg HA. (**D**): PD + 80 mg/kg HA. (**E**): PD + 160 mg/kg HA. (**F**): PD + 320 mg/kg HA.

**Figure 9:**
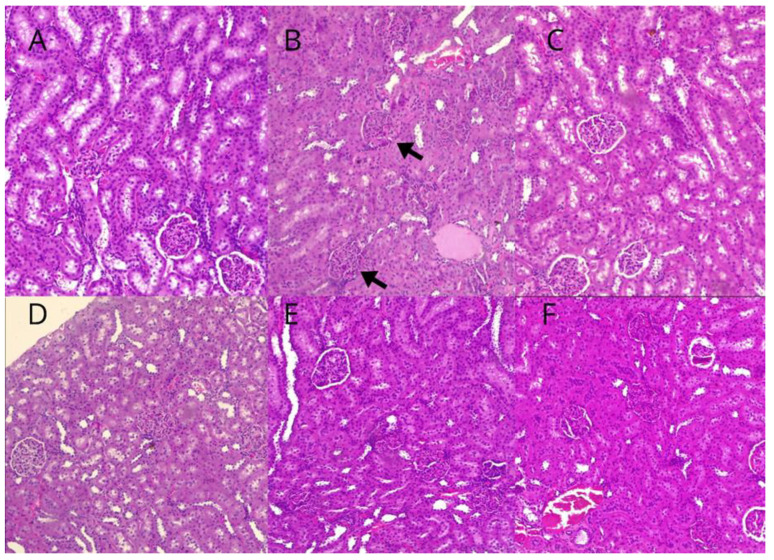
Representative images of renal tissue from the experimental groups. Magnification: 4× in optical microscopy. Arrows indicate renal lesions (congestion) with nephron involvement. (**A**): Control group. (**B**): Periodontal disease (PD) group. (**C**): PD + 40 mg/kg HA. (**D**): PD + 80 mg/kg HA. (**E**): PD + 160 mg/kg HA. (**F**): PD + 320 mg/kg HA.

Regarding the results of the histopathological evaluation of the liver, no hepatic alterations were observed in any of the experimental groups, including the animals from the control and PD groups (Table 2, Figure 8). However, renal alterations were noted in the animals in the PD group, including moderate diffuse congestion and the disappearance of Bowman’s capsule in all samples, while no changes were observed in the control group (Figure 9). In animals treated with humic acid (HA) at doses of 40 and 80 mg/kg, there was a lower frequency and severity of lesions, which were classified as mild congestion, compared to those in the PD group. Conversely, in the group treated with HA at a dose of 160 mg/kg, two animals exhibited more severe lesions, including pronounced congestion and necrosis, while two others showed mild congestion, and one animal displayed no lesions. In the group treated with 320 mg/kg of HA, one animal exhibited pronounced congestion and hydropic degeneration, while the others displayed moderate lesions or no alterations.

Based on the results, we found that HA treatment not only improved bone parameters but also offered a protective effect against renal alterations induced by PD.

## 4. Discussion

The primary findings of the present study highlight the protective effects of HA on alveolar bone, epithelial attachment loss, and osteocyte density in animals subjected to PD. Furthermore, our results indicate that treatment with HA at doses of 40 and 80 mg/kg mitigated the renal lesions induced by PD. Only three studies have assessed the effects of HA on the progression of PD [12,16,22]. In the studies by Çalışır et al. [16,22], HA extracted from the Black Sea was administered for 14 days to rats induced with PD. These studies demonstrated a reduction in ABL and improvements in both local and systemic inflammatory markers, including interleukin-1β and interleukin-10. Similarly, in the study by Orlando et al. [12], the effects of 28 days of treatment with HA derived from agricultural biomass were evaluated in rats with PD. The study also showed a reduction in ABL, along with improvements in the systemic inflammatory cytokine profile, including TNF-alpha, IL-1β, and IL-10. These investigations reported the HA treatment of ABL, consistent with our findings. To our knowledge, this is the first study to evaluate the effects of different doses of HA derived from vermicompost on the progression of periodontitis.

The protective effects of HAs on bone resorption in PD may be attributed to their anti-inflammatory and antioxidant potential, which is enhanced in compounds derived from vermicomposting [23,24]. HA possesses phenolic and carboxylic groups in its chemical structure, exhibiting chelating properties that attract and capture metallic ions, such as iron, zinc, copper, and heavy metals. This action reduces free radical formation and, consequently, oxidative stress [13,25]. These groups may also facilitate the release of hydrogen radicals by reacting with reactive oxygen species (ROS), producing more stable radicals that enhance antioxidant properties [26]. Additionally, HAs may act as prebiotic agents, promoting the growth and maintenance of beneficial microorganisms, which in turn increases the production of short-chain fatty acids that assist in generating anti-inflammatory substances [27]. It has been reported that the administration of HA extracted from the Black Sea led to a reduction in markers of gingival inflammation in animals with PD [16], potentially explaining the reduction in epithelial attachment loss observed in the animals treated with HAs in the present study. Furthermore, in a prior study conducted by our group, we noted a reduction in circulating levels of TNF-α and the TNF-α/IL-10 ratio in animals with PD treated with 80 mg/kg of HAs [12]. Although this pattern is systemic, it demonstrates the modulatory effect of this compound on the inflammatory profile in PD.

Supporting the results from the histomorphometric evaluation, our study observed the protective effects of different HA doses on the composition and topography of alveolar bone as assessed through SEM images. A similar pattern was noted in our previous study, which administered HA at a dose of 80 mg/kg [12]. To our knowledge, this is the first study to evaluate the elemental composition of alveolar bone in animals with PD treated with varying doses of HAs derived from vermicompost. Another pioneering aspect of our study was the examination of the effects of HA treatment on osteocyte density, revealing a significant increase in the number of these cells per assessed area in rats with periodontitis subjected to treatment. Osteocytes play a vital role in regulating bone metabolism and promoting remodeling [28] as they can produce osteoprotegerin (OPG), preventing RANKL from activating osteoclastogenesis [29,30]. This mechanism may explain the observed improvements in alveolar bone composition and the attenuation of bone resorption in the animals treated with HAs in the present study.

Another factor potentially related to the enhancement of bone composition is the chelating property of HA [31]. Previous studies have shown that HA can increase the activity of gastrointestinal enzymes, which are proteolytic and lipolytic, thereby impacting the absorption of minerals such as calcium and phosphorus [31]. Enhanced proteolysis leads to a greater presence of free amino acids, lysine, and arginine, preventing the formation of insoluble calcium complexes with other components, thereby improving their bioavailability [12,32]. Additionally, the increased activity of lipolytic enzymes reduces the presence of long-chain unsaturated fatty acids, which can form insoluble complexes with calcium and impair its intestinal absorption [31].

In this study, we observed no hepatic alterations in response to periodontitis or treatment with HAs. These findings are consistent with the existing literature, which has even attributed a hepato-modulatory effect to HAs in animal models of induced liver injury [33]. Conversely, in renal tissue, we observed alterations associated with periodontitis. Indeed, PD is a risk factor for the development of kidney diseases due to the increase in pro-inflammatory mediators, pro-thrombotic factors, and oxidative stress that can damage renal structures [34]. In our study, animals treated with HAs at doses of 40 and 80 mg/kg exhibited a reduction in renal lesions compared to those in the PD group. Previous studies reported positive effects of these substances in animal models of ischemic injury, where HAs reduced renal tubular dilation and damage, as well as necrosis [35]. This therapeutic effect has been attributed to the antioxidant properties of HAs [35]. However, despite the observed benefits of HAs on renal integrity at lower doses, we noted exacerbation of lesions in specific animals at doses of 160 and 320 mg/kg (one from each group). This result is intriguing given that a study administering HAs over 91 days did not report adverse effects on renal tissue [14]. Nevertheless, as HAs are heterogeneous compounds, their properties may exhibit slight variations depending on their origin, natural occurrence, or extraction procedures [13]. Thus, future studies should consider specific evaluations of renal tissue in animals treated with high doses of HAs derived from vermicompost.

Despite taking all precautions, our study is not without limitations. We did not assess local inflammatory markers or bone remodeling parameters. Future studies should consider incorporating the evaluation of RANKL (Receptor Activator of Nuclear Factor Kappa-B Ligand) and OPG (osteoprotegerin), as these markers provide valuable insights into the progression of periodontal disease (PD). Another limitation is that the use of only male animals prevents us from observing the treatment effects on females. Therefore, future studies could also evaluate the effects of HA therapy in female animals.

## 5. Conclusions

Treatment with HA significantly improved the parameters of alveolar bone in an experimental model of PD. Renal lesions induced by PD were notably attenuated, particularly at lower doses of HAs (40 and 80 mg/kg). Our findings provide valuable insights for future research, which may explore the effects of HAs derived from vermicompost on other bone diseases.

## Figures and Tables

**Table 1:** Elemental composition of the humic acid isolated from vermicompost determined by Energy Dispersive X-ray Spectroscopy (EDS).

Elements	Normalized Mass of Elements, %	Statistical Error (σ), %
Carbon	40.5	0.2
Oxygen	40.5	0.2
Sodium	10.9	0.1
Bromine	3.5	0.0
Silicon	2.0	0.0
Potassium	1.2	0.0
Calcium	0.4	0.0
Iron	0.8	0.0
Titanium	0.2	0.0

**Table 2:** Histopathological analysis of lesions in hepatic and renal tissues across different experimental groups.

Groups	Kidney						Liver					
	Animal						Animal					
	1	2	3	4	5	6	1	2	3	4	5	6
G1	+	+	+	+	+	+	+	+	+	+	+	+
G2	+++	+++	+++	+++	+++	+	+	+	+	+	+	+
G3	+++	+++	+++	++	+	+	+	+	+	+	+	+
G4	+	++	++	++	+	+	+	+	+	+	+	+
G5	+++	++++	++	+	+	++	+	+	+	+	+	+
G6	++++	++	+++	+	+	+	+	+	+	+	+	+

+: No alterations. ++: Mild alteration. +++: Moderate alteration. ++++: Severe alteration.

## Data Availability

The raw data supporting the conclusions of this article will be made available by the authors on request.
